# Comparative Transcriptome and Phytochemical Analysis Provides Insight into Triterpene Saponin Biosynthesis in Seeds and Flowers of the Tea Plant (*Camellia sinensis*)

**DOI:** 10.3390/metabo12030204

**Published:** 2022-02-24

**Authors:** Cong Chen, Huanqing Zhu, Jiaxin Kang, Hasitha Kalhari Warusawitharana, Shuna Chen, Kaixi Wang, Fei Yu, Yuanyuan Wu, Puming He, Youying Tu, Bo Li

**Affiliations:** Department of Tea Science, Zhejiang University, 866 Yuhangtang Road, Hangzhou 310058, China; 21916062@zju.edu.cn (C.C.); 22116067@zju.edu.cn (H.Z.); 22116061@zju.edu.cn (J.K.); 11816127@zju.edu.cn (H.K.W.); 22016068@zju.edu.cn (S.C.); 22016169@zju.edu.cn (K.W.); 21916126@zju.edu.cn (F.Y.); yywu@zju.edu.cn (Y.W.); pmhe@zju.edu.cn (P.H.); youytu@zju.edu.cn (Y.T.)

**Keywords:** saponin, biosynthesis, *Camellia sinensis*, seeds, flowers, transcriptome

## Abstract

Triterpene saponins exhibit various biological and pharmacological activities. However, the knowledge on saponin biosynthesis in tea plants (*Camellia sinensis* L.) is still limited. In this work, tea flower and seed samples at different developmental stages and leaves were collected and analyzed with UPLC-PDA-MS and RNA sequencing for saponin determination and transcriptome comparison. The saponin content reached around 19% in the freshly mature seeds and 7% in the green flower buds, and decreased with the fruit ripeness and flower blooming. Almost no saponins were detected in leaf samples. PCA and KEGG analysis suggested that the gene expression pattern and secondary metabolism in TF1 and TS2 vs. leaf samples were significantly different. Weighted gene coexpression network analysis (WGCNA) uncovered two modules related to saponin content. The mevalonate (MVA) instead of 2-C-methyl-d-erythritol-4-phospate (MEP) pathway was responsible for saponin accumulation in tea plants, and 3-hydroxy-3-methylglutaryl-CoA synthase (HMGS), diphosphomevalonate decarboxylase (MVD) and isopentenyl diphosphate isomerase (IDI) may be the key enzymes involved in saponin biosynthesis in tea seeds and flowers. Moreover, ten transcription factors (TFs) were predicted to regulate saponin biosynthesis in the tea plant. Taken together, our study provides a global insight into the saponin biosynthesis and accumulation in the tea plant.

## 1. Introduction

Tea is the second most popular beverage in the world after water. It is made from the leaves and buds of *Camellia sinensis* (L.) O. Kuntze, Theaceae [1]. Most research on tea has focused on its leaves and has paid less attention to the seeds and flowers. The two latter were considered to be wastes in tea gardens and were always removed from tea plants to improve leaves’ yield and quality in the next year. Recently, more and more studies revealed that tea seeds and flowers were valuable resources due to their abundant bioactive components and economic interest. Tea seeds contain saponins, fatty acids, flavonoid glycosides, phenols, proteins and starches, and are considered to be a new potential material of high-end edible oil and daily chemical supplies [2,3]. Tea flowers have similar catechin content and less caffeine compared with the leaves, and contain polysaccharides, saponins, proteins and aromatic compounds [4]. These flowers are beneficial for regulating intestinal health, immunity and obesity, and have been used as traditional medicines in Asia [5,6].

Saponins are a class of natural triterpene or steroid glycosides, which participate in plant communication and defense, and exhibit various biological activities [7]. It was found that oleanane-type triterpene saponins are abundant in tea seeds and flowers. So far, around 90 saponins have been identified in different tissues of *C. sinensis*. The numbers of known saponins in leaves, flowers, seeds and wood tissues (root, stem and bark) are 12, 24, 58 and 19, respectively. These compounds possess lots of bioactive properties, including detergence, foaming, anti-inflammation, anti-microorganism, anti-cancer, neuroprotection, gastroprotection and anti-allergy properties, and they also promote the absorption of pollutants by plants and have promising applications in agriculture, the chemical industry and medicine [8,9].

The biosynthesis of triterpene saponins has been studied in a variety of plants, such as *Panax ginseng* and *Quillaja Saponaria* [10]. The upstream biosynthetic pathways of triterpene saponins are the mevalonate (MVA) and 2-C-methyl-d-erythritol-4-phospate (MEP) pathways. The former originates from acetyl-CoA as the substrate, and the latter derives from pyruvate and glyceraldehyde 3-phosphate. Both the pathways lead to the generation of isopentenyl pyrophosphate (IPP) and dimethylallyl pyrophosphate (DMAPP), which are the common precursors of triterpenes. Then, IPP and DMAPP are catalyzed by geranyl pyrophosphate synthetase (GPS), farnesyl pyrophosphate synthetase (FPS), squalene synthase (SS) and squalene epoxidase (SE) successively, and generate 2,3-oxidosqualene. Various triterpene saponins are derived from this compound via different structural modifications, such as cyclization, hydroxylation, glycosylation by oxidosqualene cyclases (OSCs), cytochrome P450s (CYP450s) and UDP-dependent glycosyltransferases (UGTs), and so on. [11,12]. Although the common synthesis pathways of triterpene saponins have been revealed, their post-modification is highly specific and diverse in different plants. Moreover, transcription factors (TFs) and miRNA are involved in the regulation of saponin synthesis, but the detailed knowledge is still limited [13,14,15].

The theoretical basis for the synthesis and accumulation of saponins in different tissues of tea plants is still not clear. With the development of high-throughput sequencing (HTS) technologies, the synthetic pathways of many secondary metabolites in varieties of plants have been elucidated [16,17]. In this study, the saponin content of tea leaves, flowers and seeds in different growth periods was determined. Then, the typical samples with high saponin levels were selected for RNA sequencing (RNA-Seq), and gene expression profiles were compared. Furthermore, WGCNA (weighted gene coexpression network analysis) was used to screen the key modules involved in saponin synthesis, and the expression of related genes, including key enzymes and transcription factors, was predicted and analyzed. This work provides important insights into saponin synthetic biology, and reveals the mechanism of tissue-specific distribution of saponins in tea plants.

## 2. Results

### 2.1. Determination of Saponin Content in Tea Leaves, Flowers and Seeds

**Figure 1:**
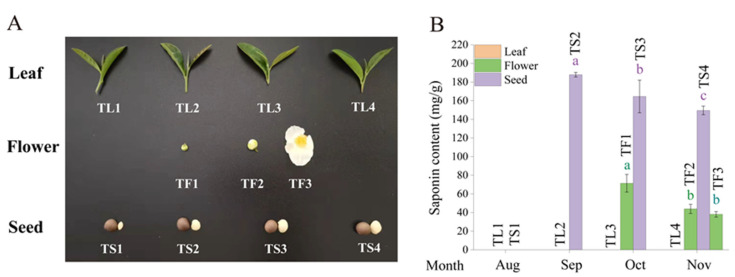
Changes of saponin content in different tissues of the tea plant. (**A**) The tea leaves, flowers and seeds were collected from September to November. (**B**) The saponin content in different samples. Data represent means ± SD from three biological replicates. Different letters indicate significant differences (*p* < 0.05).

The triterpene saponins in the aerial parts of tea plants, including leaves, flowers and seeds, were analyzed via UPLC-PDA-MS. As shown in Figure 1 and Supplemental Figure S1, almost no saponins were detected in the four leaf samples (TL1–TL4) under UV detection and mass spectrometry, while we identified eleven and twenty kinds of main saponins in flowers and fruits, respectively (Table S1). The saponin content in the green bud (TF1) reached 71.39 mg/g dry weight, and gradually decreased to 38.05 mg/g as the flowers bloomed (TF2 and TF3). For seeds, saponin was not found in the young fruits (TS1). The maximum content of tea saponins was 187.91 mg/g in the freshly mature seeds (TS2), which then declined with the fruit ripeness (TS3 and TS4). Overall, the saponin content in the three parts of tea plants can be ranked as seeds > flowers > leaves.

### 2.2. Characterization and Quality Assessment of Transcriptome Data

Eighteen sequencing libraries were generated from tea leaves (TL2), flowers (TF1, TF2) and seeds (TS1, TS2 and TS3), with three biological replicates per sample. The raw reads were 40.41–79.83 million, and 40.32–79.09 million clean reads (5.55–10.42 G clean bases) were obtained after filtration. The percentages of Q20 bases and Q30 bases were more than 97% and 92%, respectively. The GC content among the total bases was 43.55 to 47.14% (Table S2). HISAT2 was used to align the clean reads with the reference genome [18]. The percentages of total mapped reads from all samples were between 89.69% and 91.75%, and the square of the Pearson correlation coefficient (R2) among three biological replicates of each sample was 0.843–0.929 (Figure S2). These results suggested that the RNA-Seq data were credible and could be used for further analysis.

### 2.3. Differential Gene Expression Analysis

**Figure 2:**
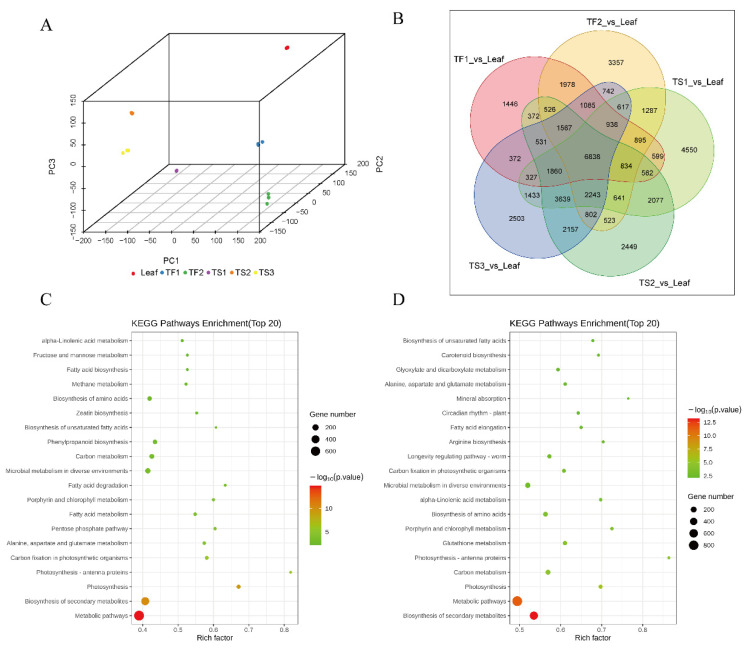
Comparative transcriptome analysis of tea leaves, flowers and seeds at different developmental stages. (**A**) Principal component analysis (PCA) of samples. (**B**) Venn diagram of DEGs in five comparison groups. (**C**) KEGG annotation of DEGs of TF1 vs. leaf. (**D**) KEGG annotation of DEGs of TS2 vs. leaf.

The six samples were separated from each other in the principal component analysis (PCA) plot, and three replicates of each sample were tightly grouped. Three seed samples at different developmental stages were closer to each other than to other samples. A similar result also occurred for the two flower samples (Figure 2A). Five pairs (TF1 vs. Leaf, TF2 vs. Leaf, TS1 vs. Leaf, TS2 vs. Leaf and TS3 vs. Leaf) were compared using the DESeq2 R package, and a Venn diagram was drawn (Figure 2B). A total of 6838 genes were shared by the five comparison groups, and 1446, 3357, 4550, 2449 and 2503 unigenes were expressed only in TF1, TF2, TS1, TS2 and TS3, respectively. 

A GO analysis showed that the DEGs between flowers and leaves (TF1 vs. Leaf) were enriched in four BP terms, including “biosynthetic process”, “organic substance biosynthetic process”, “cellular biosynthetic process” and “cellular nitrogen compound biosynthetic process” (*p* < 0.05). For the seed–leaf pair (TS2 vs. Leaf), four significantly enriched BP terms (heterocycle biosynthetic process, aromatic compound biosynthetic process, nucleobase-containing compound biosynthetic process and organic cyclic compound biosynthetic process) and two MF terms (small molecule binding, carbohydrate derivative binding) were observed (*p* < 0.05) (Figure S3). A KEGG analysis was further performed to determine the metabolic pathway of DEGs in the two comparison groups (TF1 vs. Leaf and TS2 vs. Leaf), and these DEGs were assigned to 15 and 12 significantly enriched pathways (FDR < 0.05) (Figure 2C,D). The top three pathways in the two comparison groups with the smallest *p* values were “Metabolic pathways”, “Biosynthesis of secondary metabolites” and “Photosynthesis”, indicating that the secondary metabolites in tea leaves, flowers and seeds were significantly different.

### 2.4. Weighted Gene Coexpression Network Analysis (WGCNA)

**Figure 3:**
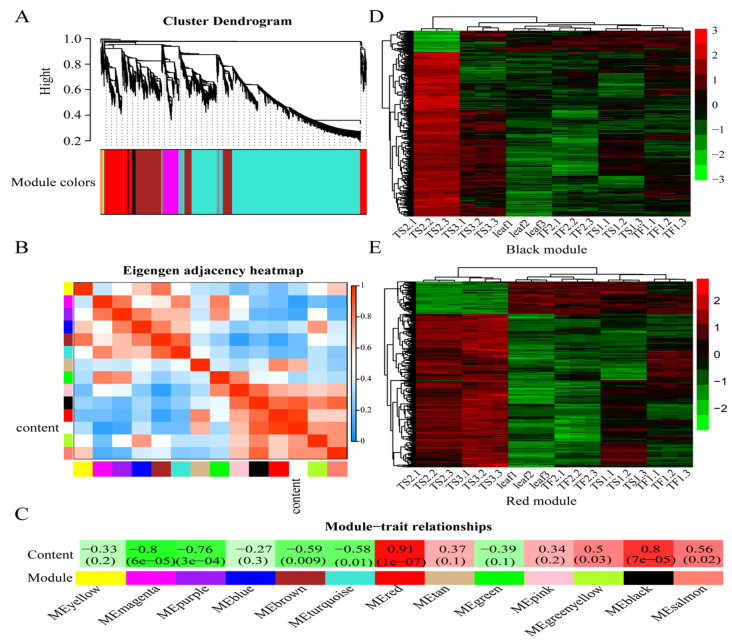
Weighted gene coexpression network analysis (WGCNA) and modules related to saponin biosynthesis. (**A**) Cluster dendrogram showing 13 coexpressed gene modules identified via WGCNA. Each module is labeled with a different color. (**B**) Eigengene adjacency heatmap showing the relationship between the modules with each other and saponin content. (**C**) Pearson correlation coefficient (R) and the *p* value for saponin content and each module. (**D**) Cluster heat map of the differentially expressed genes (DEGs) from the MEblack module. (**E**) Cluster heat map of DEGs from the MEred module. The color in the heat map represents the corresponding FPKM value of genes.

The WGCNA was performed to identify the gene sets related to saponin biosynthesis and accumulation in tea seeds and flowers. The common DEGs derived from TF1 vs. Leaf and TS2 vs. Leaf were selected, and then the genes with an FPKM value of zero were excluded. Finally, a total of 11,228 DEGs were obtained for WGCNA. Figure 3A showed that these DEGs were divided into 13 coexpressed gene modules. Subsequently, the saponin content was used as a phenotype for correlation analysis with the obtained modules. The “red” and “black” modules were significantly positively correlated with saponins, with correlation coefficients of 0.91 (*p* = 1 × 10^−7^) and 0.80 (*p* = 7 × 10^−5^), respectively (Figure 3B,C). A total of 644 and 739 genes were involved in MEred and MEblack, and their expression levels in different samples were plotted as heat maps (Figure 3D,E). Most of these genes exhibited a TS2 and TS3 preference pattern, and part of them were highly expressed in TF1 and TS1. The gene expression pattern of TF2 was similar to the leaves, but distinct from TF1, indicating that the detected saponins in TF2 were accumulated during the TF1 stage instead of in de novo synthesis.

### 2.5. Characterization of Genes Involved in Saponin Biosynthesis

**Figure 4:**
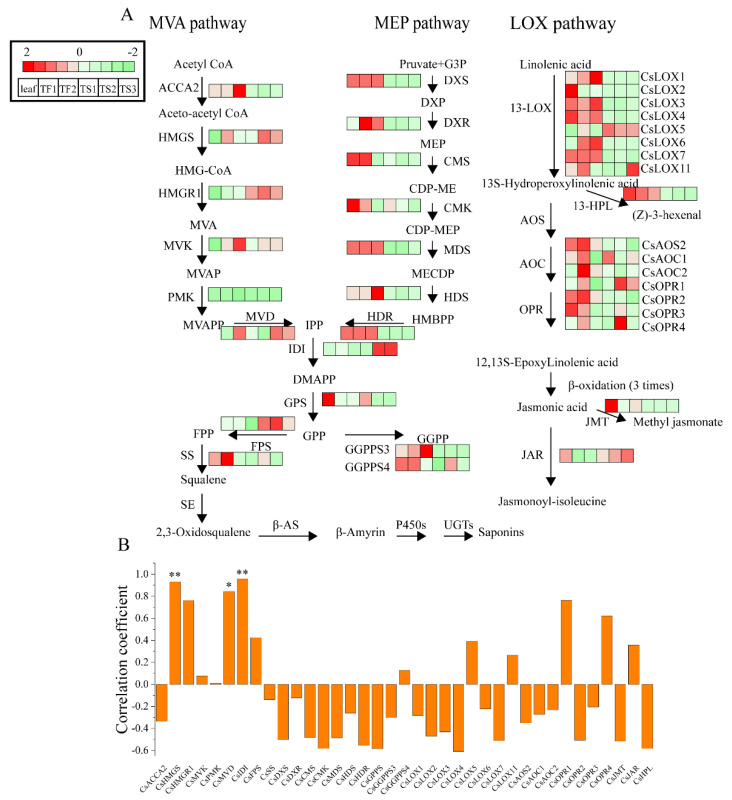
Expression pattern of genes putatively involved the biosynthesis of triterpene saponins in tea leaves, flowers and seeds. (**A**) Expression of genes in MVA, MEP and LOX pathways. (**B**) Correlation analysis between saponin content and biosynthesis-related genes. * *p* < 0.05, ** *p* < 0.01. MVA, mevalonate; MEP, 2-methyl-d-erythritol-4-phosphate; LOX, lipoxygenase; ACCT, 3-ketoacyl-CoA thiolase; HMGS, 3-hydroxy-3-methylglutaryl-CoA synthase; HMGR1, 3-hydroxy-3-methylglutaryl coenzyme A reductase; MVK, mevalonate kinase; PMK, phosphomevalonate kinase; MVD, diphosphomevalonate decarboxylase; IDI, isopentenyl diphosphate isomerase; FPS, farnesyl diphosphate; SS, squalene synthase; DXS, 1-deoxy-d-xylulose-5-phosphate synthase; DXR, 1-deoxy-d-xylulose 5-phosphate reductoisomerase; CMS, 2-C-methyl-d-erythritol 4-phosphate cytidylyltransferase; CMK, 4-diphosphocytidyl-2-C-methyl-d-erythritol kinase; MDS, 2-C-methyl-d-erythritol 2,4-cyclodiphosphate synthase; HDS, hydroxymethylbutenyl diphosphate synthase; HDR, hydroxymethylbutenyl diphosphate reductase; GPPS, geranyl pyrophosphate synthase; GGPPS3, geranylgeranyl pyrophosphate synthase 3; GGPPS4, geranylgeranyl pyrophosphate synthase 4; 13-LOX, 13-lipoxygenase; AOS, allene oxide synthase; AOC, allene oxide cyclase; OPR, 12-oxo-phytodienoic acid reductase; JMT, jasmonic acid carboxyl methyltransferase; JAR, jasmonic acid amido synthetase; 13-HPL, 13-hydroperoxide lyase.

Based on the WGCNA result, the genes involved in the mevalonate (MVA) pathway, 2-C-methyl-d-erythritol-4-phospate (MEP) pathway and lipoxygenase (LOX) pathway were further analyzed, and their expressions in different samples are shown in Figure 4A and Supplemental Table S3. For the MVA pathway, 3-hydroxy-3-methylglutaryl-CoA synthase (*HMGS*), mevalonate kinase (*MVK*) and diphosphomevalonate decarboxylase (*MVD*) were highly expressed in flower and seed samples with more saponins. However, higher mRNA levels of 3-hydroxy-3-methylglutaryl coenzyme A reductase (*HMGR1*), isopentenyl diphosphate isomerase (*IDI*) and farnesyl diphosphate synthase (*FPS*) were only observed in tea seeds. For the MEP pathway, most of the genes were highly expressed in the leaves and flowers compared with the seeds, except for 1-deoxy-d-xylulose 5-phosphate reductoisomerase (*DXR*) which exhibited the most abundant expression in flowers. These results suggested that the triterpene saponins were biosynthesized mainly through the MVA pathway in seeds and flowers.

Jasmonic acid derivatives, including jasmonoyl-isoleucine (JA-ile) and methyl jasmonate (MeJA), are known to promote triterpene saponin accumulation in various plants [19]. Therefore, next we analyzed the key genes in the jasmonic acid (JA) pathway. As shown in Figure 4A, the gene expression patterns of enzymes that catalyze the synthesis of JA, MeJA and JA-ile were significantly different in leaves, flowers and seeds. Three genes, namely, 13-lipoxygenase 5 (*LOX5*), 12-oxo-phytodienoic acid reductase 1 (*OPR1*) and *OPR4*, exhibited higher expression intensities in seeds and flowers compared with leaves, and the specific highest expression of *LOX6* and *AOC2* were observed in flowers.

A correlation analysis between tea saponin content and the transcriptional abundance of key genes involving the MVA, MEP and JA pathways was further conducted. As shown in Figure 4B, the saponin content was highly correlated with the expression of *CsHMGS*, *CsMVD* and *CsIDI* (*p* < 0.05 or *p* < 0.01), indicating that the MVA pathway played a vital role in the synthesis of tea saponins.

### 2.6. Characterization of Hub TFs Involved in Saponin Biosynthesis

**Figure 5:**
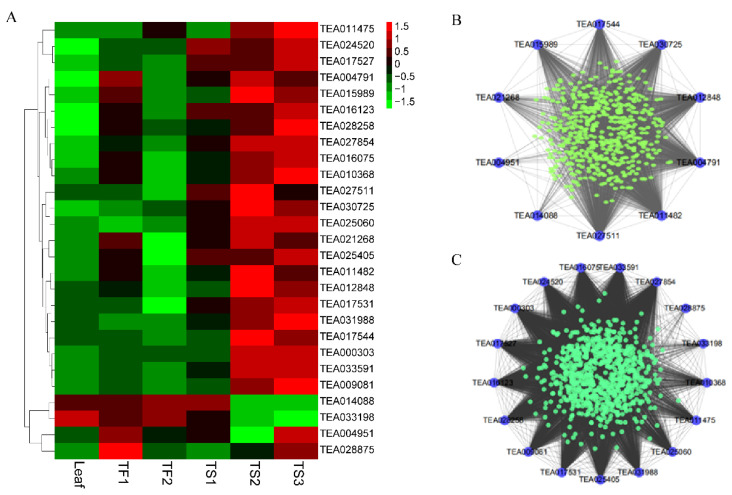
Analysis of hub TFs in black and red modules. (**A**) Heatmap displaying the expression patterns of all hub TFs in different samples. (**B**) The transcriptional regulatory network of the hub TFs in the black module. (**C**) The transcriptional regulatory network of the hub TFs in the red module.

A total of 27 transcription factors (TF) were found in the red and black modules, and their expression patterns are presented in Figure 5A. Most of these TFs were highly expressed in the mature seeds (TS2 and TS3), and lowly expressed in leaves. Cytoscape was used to represent the interaction network diagram of these TFs with other genes in each module (Figure 5B,C). Table 1 showed that these TFs belonged to nine TF families, including AP2, bHLH, bZIP, GARP, GATA, MYB, TCP, Trihelix and WRKY. Among them, the expressions of eight TFs (TEA004791, TEA012848, TEA017531, TEA016075, TEA011482, TEA015989, TEA027854 and TEA009081) were positively correlated with the saponin content in both the seeds and flowers, and two TFs (TEA014088, TEA033198) were negatively related (*p* < 0.05 or *p* < 0.01). Some of the other TFs were only associated with the saponin content in either flowers or seeds (*p* < 0.05 or *p* < 0.01). The interaction network diagram analyzed with Cytoscape showed that the above-mentioned TFs were highly coexpressed with *HMGS*, *HGMR1*, *CsIDI*, *CsMVD* or *CsOPR4*, which further illustrated that they were very relevant to tea saponin synthesis.

### 2.7. qRT-PCR Analysis

**Figure 6:**
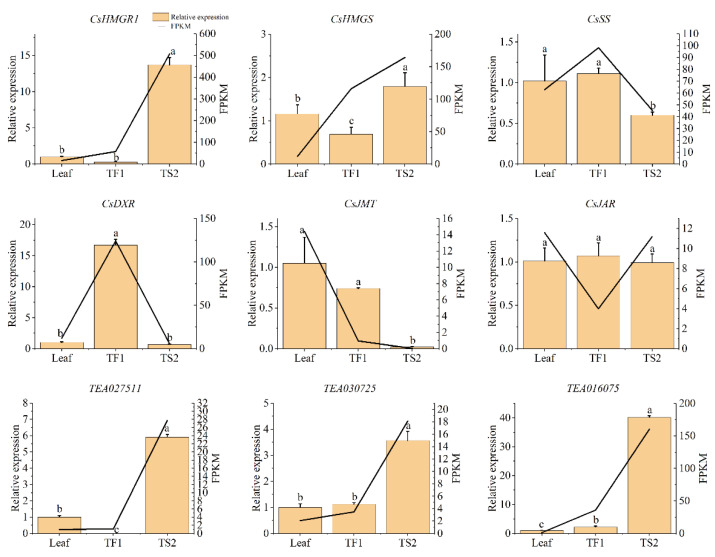
Relative expression of nine selected genes involved in saponin metabolism to EF-1α. Data represent means ± SD from three biological replicates. Different letters indicate a significant difference (*p* < 0.05). The column represents the gene mRNA level determined with qRT-PCR. The line represents the FPKM value of the gene.

Considering that the bHLH family have been reported to regulate triterpene synthesis, three predicted bHLH members (*TEA016075*, *TEA027511* and *TEA030725*) and six structural genes (*HMGS*, *HGMR1*, *SS*, *DXR*, *JMT* and *JAR*) involved in saponin biosynthesis were determined via qRT-PCR, in order to verify the accuracy of the RNA-Seq data. As shown in Figure 6, the expressions of most genes were basically consistent with the transcriptome data. *HMGS*, *HGMR1* and all three selected TFs were highly expressed in seeds (TS2), and the highest mRNA expression of *DXR* was observed in flowers (TF1). These genes might contribute to the saponin accumulation in tea seeds and flowers. The expressions of *JMT* and *JAR* were not higher in flowers and seeds, which verified that the JA pathway did not participate in tea saponin biosynthesis.

## 3. Discussion

Saponins, as a class of widely distributed natural compounds with various biological activities, have been found in more than 100 families of plants and a few marine organisms, such as *Theaceae*, *Asparagaceae*, *Liliaceae*, *Agavaceae*, *Solanaceae*, *Alliaceae*, *Poaceae* and starfish, etc. [13,20,21,22,23]. The tea seeds and flowers are rich in triterpene saponins, and their chemical structures and some bioactivities have been reviewed [8,9,24]. Although tea saponins have aroused a lot of interest due to various potential applications in daily chemicals, agriculture, food and medicine, their biosynthesis in the tea plant remains unclear.

In this study, we first compared the saponin content in tea leaves, flowers and seeds at different stages. The total saponin content in seeds was more than twice that in flowers, and the content reached approximately 19% and 7%, respectively. Almost no saponins were detected in leaves (Figure 1). The green bud contained higher saponin content than white bud and full bloom, which was in accordance with the previous report on the content change of saponins in different developmental stages of tea flowers [25]. Whether the foam in tea soup is related to saponins is still uncertain, because little is known about the saponins in tea leaves. A recent study showed that saponin content in the buds and leaves from different tea cultivars varied from 0.48 to 3.48 mg/g dry weight, and were relatively stable during the tea processing [24]. The quantification of saponins from tea plants is still a challenge due to their complex composition and a lack of standards, although a few UPLC-PDA-MS/MS methods have been developed [26]. A low response to UV and MS detectors and inefficient separation are the major limitations for the determination of trace saponin compounds in tea plants. Anyway, the existing results suggested that the saponins in tea plants were mainly distributed in seeds and flowers, rather than in leaves. 

Saponins are usually distributed in plants in tissue-specific and development-dependent manners, which may be helpful to defend against pests and pathogens. For instance, saponin biosynthesis in the leaves of *Paris polyphylla var. yunnanensis* (Melanthiaceae) and *Panax notoginseng* (Araliaceae) is stronger than that in rhizomes during the vegetative stage, but this is completely the opposite at the fruiting stage [27,28,29]. The ginsenosides are concentrated in roots instead of the flowers of Panax ginseng [30]. Compared with tea seeds, the saponin content in the seeds of other plants is very low [31,32]. It is around 1.1–1.52% in wild soybean (*Glycine soja Sieb et Zucc.*), 0.1–5% in seeds of *Chenopodium quinoa* and 0.48–0.7% in seeds of *Tribulus terrestris* [33]. Tea seeds are a potential natural resource for the preparation of triterpene saponins due to their low cost and large yield.

In order to explore the enrichment mechanism of saponins in the tea plant, the transcriptome of tea leaves, flowers and seeds at different growth periods was analyzed. The data showed that the secondary metabolism in different organs was remarkably diverse (Figure 2), and two modules containing 1383 DEGs were found to be positively correlated with saponin content (Figure 3). MVA and MEP are two classic pathways involved in the biosynthesis of plant triterpene saponins [13]. In this work, several key enzyme genes in the MVA pathway were highly expressed in both the seeds and flowers compared with leaves, especially *HMGS*, *MVD* and *IDI*, which were significantly correlated with the saponin content (Figure 4). These results suggested that saponins are mainly synthesized via the MVA pathway in tea plants and explained the low saponin content in tea leaves. Previous studies showed that the MVA pathway played a leading role in the biosynthesis of triterpene saponins in many plants. The biosynthesis of triterpene saponins in ginseng, Panax notoginseng and some other plants can be regulated by controlling the expression of *HMGR*, *FPS*, *SS* and *SE* [14,15]. The MEP pathway usually contributes to the biosynthesis of monoterpenoids and diterpenoids [34,35]. The transcription levels of MEP pathway-related genes in leaves and flowers were higher than that in seeds, which explained the enrichment of terpenoid aroma chemicals in the two tissues. The addition of exogenous JA and its derivatives, such as MeJA and methyl dihydrojasmonate (MDJ), or the stimulation of JA biosynthesis, have been shown to promote triterpenoid saponin production and the upregulation of saponin biosynthesis-related genes in several plants, including *Panax ginseng*, *Panax notoginseng* and *Calendula officinalis* [12,36,37]. In this study, most of the JA synthesis-related genes were highly expressed in leaves and flowers, and none of them were significantly associated with saponin content (Figure 4). This result suggested that the JA pathway did not play a key role in the biosynthesis of saponins in tea plants.

Transcription factors (TFs) are a class of DNA-binding proteins that interact with gene promoters, and which widely regulate plant growth and secondary metabolism [38]. Until now, the TFs involved in triterpene biosynthesis are mainly distributed in bHLH (basic helix–loop–helix), AP2/ERF (Apetala2/ethylene response factor), bZIP (basic region/leucine zipper motif) and WRKY families, and the bHLH members in different plants, such as *Medicago truncatula*, *Cucumis sativus* and *Panax notoginseng*, were mostly reported [15]. For instance, two jasmonate-inducible bHLH TFs, namely, TSAR1 and TSAR2, could coregulate *HMGR1* and an E3 ubiquitin ligase gene Makibishi1, which controlled HMGR1 levels and increased the accumulation of triterpene saponin biosynthesis in Medicago truncatula. Both TFs transactivated *HMGR1* via binding to the N-box in its promoter, but exhibited different regulation patterns of downstream saponin biosynthetic genes [39]. An ERF TF (PjERF1) from *Panax japonicus* were proven to bind with the promoters of *βAS*, *CAS* and *SE*, and regulated triterpene saponin biosynthesis [40]. A bZIP TF gene *BcbZIP134* negatively regulated the biosynthesis of saikosaponins in the roots of Chaihu [41]. In this study, a total of 27 TFs were obtained after WGCNA analysis. Among them, the expression of eight and two TFs were positively and negatively related, respectively, with the saponin content in both the seeds and flowers. These TFs might control saponin biosynthesis by transcriptionally regulating *HMGR1*, *HMGS*, *MVD* and *IDI*. So far, the knowledge on the TFs related to saponin biosynthesis in plants are still limited. More studies are needed to further explore the regulatory network of plant saponin metabolism, and will help the development of technology for the artificial synthesis of saponins.

## 4. Materials and Methods

### 4.1. Plant Materials

The tea leaves, flowers and seeds in different periods were collected from *Camellia sinensis var. sinensis* ‘Jinxuan’, which was cultivated in the tea garden of Zhejiang University (Hangzhou, China). The first and second leaves were plucked in every month from August to November 2020 and named TL1 to TL4. Three flower samples, including green bud (in October, TF1), white bud (in November, TF2) and full flower (in November, TF3), and four seed samples, including young seeds (in August, TS1), freshly mature seeds (in September, TS2), mature seeds (in October, TS3) and old seeds (in November, TS4), were collected during the same period. All the samples were frozen in liquid nitrogen after harvest, and then stored in at −80 °C until use.

### 4.2. Determination of Triterpene Saponins by UPLC-PDA-MS

The leaves, flowers and seeds were lyophilized and milled into powder. The samples were extracted with 70% methanol (1:15, *w*/*v*) at 70 °C for 30 min, and the supernatant was collected after centrifugation at 12,000× *g* for 5 min. The saponins were determined using the ACQUITY UPLC/MS system equipped with a PDA detector (Waters, Milford, MA, USA) and an electrospray ionization (ESI) source (Waters, Milford, MA, USA) using a method developed in our lab [26]. Briefly, a 5.0 μL sample was injected and eluted on a Waters Acquity UPLC HSS T3 column (1.8 µm, 150 mm × 2.1 mm i.d., Waters, Milford, MA, USA) at a flow rate of 0.2 mL/min. The mobile phase A and B were water and acetonitrile, both containing 0.1% formic acid. The gradient elution was as follows: 0–4 min, 35–37% B; 4–32 min 37% B; 32–58 min, 37–45% B; 58–60 min, 35% B. The detection wavelength was set at 210 nm, and the column temperature was 30 °C. For mass spectrometry (MS) analysis, the conditions were: negative ion mode; scan range, *m*/*z* 100–2000; capillary voltage, 3 kV; cone voltage, 40 V; extractor voltage, 4 V; source temperature, 150 °C; desolvation temperature, 350 °C; cone gas flow, 50 L/h; desolvation gas flow, 600 L/h. Theasaponin E1 (purity 98.0%) was prepared as previously described and used as the external standard for saponin quantification [26]. The content of saponins was calculated by bringing the area of each individual peak under the PDA detector into the standard curve of theasaponin E1 (Supplemental Table S4).

### 4.3. RNA-Seq Analysis

One leaf sample (TL2), two flower samples (TF1 and TF2) and three seed samples (TS1, TS2 and TS3) were chosen for RNA-Seq analysis in three biological replicates. Total RNA was extracted using an RNAprep pure plant kit (Tiangen Biotech Co., Ltd., Beijing, China). RNA integrity, concentration and quality were evaluated with an Agilent 4150 TapeStation (Agilent Technologies, Palo Alto, CA, USA). The cDNA library of each sample was constructed and sequenced on an Illumina Hiseq platform (Illumina, San Diego, CA, USA). The raw data were filtered using Fast QC software, and the clean reads were mapped onto the Tea Plant Information Archive (TPIA: http://tpia.teaplant.org/, accessed on 2 December 2020) using HISAT2 (v 2.2.1) [18,42]. The fragments per kilobase of the transcript per million fragments mapped (FPKM) values were used to indicate gene expression levels. Differentially expressed genes (DEGs) were analyzed using the DESeq2 software, and the screening criteria for DEGs were the adjusted *p* value (padj) < 0.05 and fold change (FC) ≥ 2 (|log 2 fold change| ≥ 1) [43]. Moreover, the identified DEGs were analyzed based on the Gene Ontology (GO) gene set (http://www.geneontology.org/, accessed on 2 December 2020) and the Kyoto Encyclopedia of Genes and Genomes (KEGG) database (http://www.kegg.jp/, accessed on 2 December 2020).

### 4.4. Weighted Gene Coexpression Network Aanalysis (WGCNA)

The coexpression of genes was analyzed using the WGCNA R package. The topological overlap measurement (TOM) was obtained through the conversion of the adjacency matrix (a power β of 12) based on the Pearson’s correlation between the selected DEGs. Then, these DEGs were clustered hierarchically by the DynamicTreeCut algorithm, and each cluster was called a module [44]. In order to screen the modules related to saponin synthesis, Pearson’s correlation between the eigengenes of each module and the saponin content were further analyzed. Cytoscape (v 3.6.0) was used to visualize the coexpression network of the selected genes [45].

### 4.5. Quantitative Real-Time PCR (qRT-PCR) Analysis

Total RNA was extracted using an RNAprep Pure Plant Plus Kit (Tiangen Biotech Co., Ltd., Beijing, China), and reverse transcribed into cDNA with an Evo M-ML V RT Premix Kit (Accurate Biology, Changsha, China) according to the operation manual. The qRT-PCR was performed on a Roche LightCycle 96 PCR System (Roche Applied Science, Mannheim, Germany) using a One Step SYBR PrimeScript PLUS RT-PCR Kit (TaKaRa Bio, Kyoto, Japan). Relative mRNA expression was analyzed with the comparative Ct method (2^−ΔΔCT^) and normalized to elongation factor-1α (EF-1α) with *C. sinensis* as an internal reference gene. The used primers were synthesized by Shenggong Bioengineering Co., Ltd. (Shanghai, China), and their sequences are listed in the Supplemental Table S5.

### 4.6. Statistical Analysis

Three biological replicates were prepared and analyzed in this work, and data were presented as means ± standard deviations (SD). Multiple comparison (Student–Newman–Keuls test) and Pearson correlation analysis were performed in SPSS Statistics 26.0 (SPSS Inc., Chicago, IL, USA). Values of *p* < 0.05 and *p* < 0.01 were considered to be significant differences and extremely significant differences, respectively.

## 5. Conclusions

In summary, we compared the saponin content in tea leaves, flowers and seeds at different developmental stages, and analyzed the transcriptome of typical samples. The saponin content in tea seeds was much higher than that in tea flowers. Freshly mature seeds and green flower bud were the most saponin-enriched tissues among the seed and flower samples, respectively. The triterpene saponins in tea plants were mainly synthetized through the MVA pathway, and CsHMGS, CsMVD and CsIDI might be key enzymes involved in saponin biosynthesis. Ten TFs were predicted to be associated with saponin accumulation in both seeds and flowers by WGCNA, and deserve to be further investigated as key regulators of saponin metabolism in the tea plant. This study provided a global understanding of saponin biosynthesis and accumulation in the tea plant.

## Figures and Tables

**Table 1:** Transcription factors in red and black modules and their correlation with saponin content and synthesis-related genes.

Family	Gene ID in CSS	R with Flower Saponin Content	R with Seed Saponin Content	Connected Genes Involving Saponin Synthesis
AP2	TEA000303	0.168	0.984 **	
	TEA004791	0.860 *	0.742 *	CsHMGR1
	TEA010368	0.717	0.762 *	CsHMGS, CsIDI
	TEA012848	0.885 *	0.882 **	CsHMGR1
	TEA014088	−0.878 *	−0.991 **	CsHMGR1
	TEA017527	0.381	0.275	
	TEA017531	0.904 *	0.844 **	CsHMGS
bHLH	TEA016075	0.889 *	0.935 **	
	TEA027511	0.953 **	0.513	CsHMGR1
	TEA030725	−0.890 *	0.818 **	CsHMGR1
	TEA033198	−0.914 *	−0.975 **	CsHMGS, CsMVD, CsIDI
bZIP	TEA011482	0.896 *	0.831 **	CsHMGR1
	TEA015989	0.884 *	0.907 **	CsHMGR1, CsOPR4
	TEA027854	0.860 *	0.892 **	
GARP	TEA011475	−0.333	0.783 *	CsHMGS, CsIDI
GATA	TEA016123	0.938 **	0.542	
MYB	TEA009081	0.845 *	0.815 **	CsHMGS
	TEA025060	−0.577	0.894 **	CsHMGS, CsIDI
	TEA025405	0.891 *	0.644	CsHMGS
	TEA028258	0.845 *	0.555	
	TEA028875	0.897 *	0.549	
	TEA031988	0.600	0.780 *	CsHMGS
TCP	TEA033591	0.694	0.971 **	
	TEA024520	0.309	0.149	
Trihelix	TEA004951	0.832 *	−0.184	CsOPR4
	TEA021268	0.952 **	0.649	CsOPR4, CsHMGR1
WRKY	TEA017544	0.521	0.842 **	CsOPR4, CsHMGR1

R: Correlation coefficient. * *p* < 0.05. ** *p* < 0.01.

## Data Availability

Data is contained within the article and Supplementary Materials.

## Supplementary Materials

The following supporting information can be downloaded at: https://www.mdpi.com/article/10.3390/metabo12030204/s1, Figure S1: Saponins from the leaves, flowers and seeds of Camellia sinensis analyzed via UPLC-PDA-QTOF-MS, Figure S2: Pearson correlation of gene expression among samples, Figure S3: Gene Ontology (GO) analysis of differentially expressed genes (DEGs) in two comparison groups, including TF1 vs. Leaf and TS2 vs. Leaf, Table S1: Saponins detected in flowers and seeds of *C. sinensis* via UPLC-PDA-QTOF-MS. Table S2: Summary of the quality and output of the RNA-Seq data, Table S3: Expression pattern of genes related to MVA, MEP and LOX pathways, Table S4: The standard curve of theasaponin E1, Table S5: Primer sequences for quantitative real-time PCR.
