# Supplementary material for: Comparative Transcriptome and Phytochemical Analysis Provides Insight into Triterpene Saponin Biosynthesis in Seeds and Flowers of the Tea Plant (Camellia sinensis)
Source: Metabolites. 2022 Feb 24;12(3):204. doi: 10.3390/metabo12030204 (PMC8949954; doi:10.3390/metabo12030204)
Supplement: Supplementary file 1 [file metabolites-12-00204-s001.zip › metabolites-1598116-supplementary.pdf]

Supplementary Table S1. Saponins detected in flowers and seeds of *C. sinensis* by UPLC-PDA-QTOF-MS.

Organ	Peak	Retention Time (min)	[M-H] - (*m*/*z*)	Formula	Putative saponins	Reference
Flower	1	25.37	1231	$C_{59}H_{92}O_{27}$	Theasaponin A2	[25]
	2	30.96	1229	$C_{60}H_{94}O_{26}$	Floratheasaponin D	
	3	32.3	1215	$C_{59}H_{92}O_{26}$	Chakasaponin I	
	4	42.24	1171	$C_{62}H_{96}O_{27}$	Floratheasaponin H	
	5	43.05	1157		New	
	6	51.31	1285	$C_{63}H_{98}O_{27}$	Chakasaponin V/ Floratheasaponin E	
	7	52.14	1271	$C_{62}H_{96}O_{27}$	Chakasaponin II	
	8	53.88	1285	$C_{63}H_{98}O_{27}$	Chakasaponin V Floratheasaponin E	
	9	54.62	1271	$C_{62}H_{96}O_{27}$	Floratheasaponin J	
	10	55.02	1285	$C_{63}H_{98}O_{27}$	Chakasaponin V/ Floratheasaponin E	
	11	55.68	1273	$C_{62}H_{98}O_{27}$	Floratheasaponin C	
Fruit	1	14.87	1187		New	[26]
	2	16.22	1217	$C_{58}H_{90}O_{27}$	Theasaponin E3/E6	
	3	20.48	1229	$C_{59}H_{90}O_{27}$	Theasaponin E1/E7	
	4	21.62	1173	$C_{57}H_{90}O_{25}$	Theasaponin C1	
	5	22.74	1273	$C_{61}H_{94}O_{28}$	Theasaponin A3	
	6	24.91	1229	$C_{59}H_{90}O_{27}$	Theasaponins E4/E8	
	7	25.77	1173	$C_{57}H_{90}O_{25}$	Theasaponin B5	
	8	26.66	1259	$C_{60}H_{92}O_{28}$	Assamsaponin H	
	9	28.66	1273	$C_{61}H_{94}O_{28}$	Theasaponin E10	
	10	29.303	1201	$C_{58}H_{90}O_{26}$	Camelliasaponin B1/B2	
	11	30.54	1301	$C_{62}H_{94}O_{29}$	Assamsaponin F	
	12	31.81	1171	$C_{57}H_{88}O_{25}$	Assamsaponin A/E	
	13	33.37	1271	$C_{61}H_{92}O_{28}$	Assamsaponin B	
	14	36.3	1229	$C_{59}H_{90}O_{27}$	Theasaponin E2	
	15	40.65	1187	$C_{58}H_{92}O_{25}$	Camelliasaponin A1/A2	
	16	42.3	1157		New	
	17	42.7	1257	$C_{61}H_{94}O_{27}$	Foliatheasaponin I/III	
	18	49.2	1285	$C_{62}H_{94}O_{28}$	Teaseedsaponin L	
	19	50.04	1185	$C_{58}H_{90}O_{25}$	Teaseedsaponin G	
	20	58.22	1271	$C_{62}H_{96}O_{27}$	Teaseedsaponin E	

Supplementary Table S2. Summary of the quality and output of the RNA-Seq data

Sample	Raw_reads	Clean_reads	Clean_bases	Q20(%)	Q30(%)	GC(%)
leaf-1	79832696	79094450	10.42G	97.92	93.92	46.54
leaf-2	62861058	62155632	8.16G	97.5	92.99	46.45
leaf-3	70375808	69696696	9.17G	97.91	93.87	46.35
TF1-1	46160438	45865230	6.35G	97.65	92.97	43.55
TF1-2	49086870	48799792	6.77G	97.55	92.69	43.57
TF1-3	40412442	40171884	5.57G	97.62	92.87	43.7
TF2-1	44481480	44187540	6.13G	97.65	92.98	44.35
TF2-2	42463132	42174196	5.85G	97.66	93.03	44.44
TF2-3	40624918	40323368	5.59G	97.67	93	44.96
TS1-1	78635468	77847198	10.37G	97.61	93.14	44.15
TS1-2	70506508	69759976	9.25G	97.61	93.12	44.14
TS1-3	67145340	66409408	8.79G	97.63	93.2	44.15
TS2-1	66826870	65353714	8.66G	97.38	92.81	47
TS2-2	61613918	60060960	7.93G	97.47	92.99	46.97
TS2-3	56991284	55825500	7.45G	97.72	93.57	47.14
TS3-1	42120088	41894586	5.81G	97.71	93.15	46.75
TS3-2	40322838	40062220	5.55G	97.62	92.96	46.65
TS3-3	42889624	42599718	5.91G	97.49	92.67	46.81

Supplementary Table S3. Expression pattern of genes related to MVA, MEP and LOX pathways

Abbreviation	GenBank accession	Gene ID in CSS	Annotation	Leaf	Flower		Seed		
				TL2	TF1	TF2	TS1	TS2	TS3
ACCA2	MH125245	TEA015906	3-Ketoacyl-CoA thiolase	9.6	10.56	33.68	4.06	2.89	4.58
HMGS	MH125246	TEA014739	3-Hydroxy-3-methylglutaryl-CoA synthase	11.79	116	58.84	48.32	164.04	128.18
HMGR1	MH125248	TEA028769	3-Hydroxy-3-methylglutaryl coenzyme A reductase	16.1	57.47	112.76	259.44	506.91	313.56
MVK	MH125249	TEA030674	Mevalonate kinase	3	24.95	58.31	14.63	22.32	19.23
PMK	MH125250	TEA022400	Phosphomevalonate kinase	4.45	5.39	2.39	6.45	5	4.87
MVD	MH125251	TEA026311	Diphosphomevalonate decarboxylase	16.72	77.9	37.5	12.62	94.09	58.46
IDI	MH125259	TEA030524	Isopentenyl diphosphate isomerase	23.29	44.49	21.96	19.19	133.89	149.8
FPS	MH125267	TEA003988	Farnesyl diphosphate synthase	49.57	42.5	19.08	87.4	110.5	58.95
SS	MF774226	CSS0031155	Squalene synthase	62.91	98.27	35.44	25.9	45.13	26.07
DXS	MH125252	TEA008876	1-Deoxy-D-xylulose-5-phosphate synthase	9.92	11.88	11.74	0.01	0.02	0
DXR	MH125253	TEA005817	1-Deoxy-D-xylulose 5-phosphate reductoisomerase	12.21	124.11	42.96	6.16	5.77	8.81
CMS	MH125254	TEA026482	2-C-Methyl-D-erythritol 4-phosphate cytidylyltransferase	15.7	17.22	5.18	8.3	4.53	6.88
CMK	MH125255	TEA033157	4-Diphosphocytidyl-2-C-methyl-D-erythritol kinase	6.72	4.65	2.4	4.21	3.34	2.45
MDS	MH125256	TEA027323	2-C-Methyl-d-erythritol 2,4-cyclodiphosphate synthase	36.78	33.99	36.43	8.13	8.41	14.64
HDS	MH125257	TEA013763	Hydroxymethylbutenyl diphosphate synthase	38.71	49.2	365.02	17.34	11.59	14.06

HDR	MH125258	TEA012305	Hydroxymethylbutenyl diphosphate reductase	98.52	87.37	92.02	6.05	5.81	6.23
GPPS	MH125260	TEA022209	Geranyl pyrophosphate synthase	127.91	11.94	8.72	23.16	3.78	2.38
GGPPS3	MH125281	TEA018068	Geranylgeranyl pyrophosphate synthase3	3.43	6.42	22.33	0.02	0.09	0.03
GGPPS4	MH125282	TEA019181	Geranylgeranyl pyrophosphate synthase4	44.22	48.67	24.36	9.24	38.61	21.43
LOX1	EU195885.2	TEA025499	13-lipoxygenase 1	2.68	4.3	22.15	0	0	0.01
LOX2	THG22978	TEA009423	13-lipoxygenase 2	235.84	0.25	0.86	0.08	0.15	0.29
LOX3	FJ794853	Novel.15329	13-lipoxygenase 3	19.92	10.61	46.38	0.2	0.03	0.5
LOX4	THG16242	TEA011776	13-lipoxygenase 4	83.15	18.14	42.2	2.34	0.13	1.39
LOX5	THG21558	TEA020304	13-lipoxygenase 5	8.39	16.04	10.28	23.74	19.78	20.08
LOX6	THG23915	TEA020832	13-lipoxygenase 6	0.13	337.77	739.77	0	0	0.01
LOX7	THG08251	TEA012289	13-lipoxygenase 7	27.05	30.67	24.94	2.92	3.43	2.23
LOX11	THG95893	TEA027370	13-lipoxygenase 11	0.86	1.57	0.39	0.15	0.01	2.28
AOS2	AHY03308.1	TEA027984	Allene oxide synthase 2	28.83	49.62	10.89	5.48	5.73	0.83
AOC1	THG219730	TEA012644	Allene oxide cyclase 1	62.56	96.31	28.2	95.79	50.62	62.84
AOC2	THG13397	TEA020075	Allene oxide cyclase 2	1.65	6.21	2.71	2.23	0.96	1.81
OPR1	THG06104	TEA025907	12-oxo-phytodienoic acid reductase 1	3.42	4.27	1.67	2.99	5.95	4.38
OPR2	THG97921	TEA029800	12-oxo-phytodienoic acid reductase 2	18.04	22.82	12.43	7.42	6.57	3.67
OPR3	THG95859	TEA027519	12-oxo-phytodienoic acid reductase 3	14.72	10.43	5.69	6.83	9.66	6.97
OPR4	THG95772	TEA026804	12-oxo-phytodienoic acid reductase 4	2.21	4.79	0.94	0.31	12.43	0.73
JMT	QNG98966	TEA031418	Jasmonic acid carboxyl methyltransferase	14.43	0.94	1.89	0.02	0.03	0.02
JAR	LOC114260773	TEA009648	Jasmonic acid amido synthetase	11.59	3.98	5.41	10.07	11.18	14.36
13-HPL	THG13575	TEA008699	13-hydroperoxide lyase	160.44	93.03	51.49	12.08	5.27	6.76

* The data indicates the mean FPKM value of the three replicates.

Supplementary Table S4. The standard curve of Theasaponin E1.

Compound	Standard curve	Concentrations (μg/mL)	R^2
Theasaponin E1	Y=125.46 X + 689.07	0-1000 μg/mL	0.99

* Y and X indicate the peak area and concentration of theasaponin E1, respectively.

Supplementary Table S5. Primer sequences for quantitative real-time PCR

Gene	Forward primer (5′-3′)	Reverse primer (5′-3′)
CsJAR	CCACAACTCCACTCCAGAAC	TGGCTCGTGAAGTCAACAAC
CsHMGR1	CTCTTCCTCCTCCTCCTCCT	CCTTTGTGCCCTTGGATAGT
CsHMGS	ATTGGTCGGCTGGAAGTGG	CATAGCAAGCATTGGTTGAGTC
CsDXR	GGCACCATGACAGGTGTTCT	GGAAGGTGCAGTCACCAACT
CsSS	CAGGAGGCAATTGAGGACAT	GATGCCAAATCTTCAGACCC
CsJMT	CGCCACAAACCACCGTAT	CAGCCTGCCGTAGAAAGAAC
CsTEA027511	TTGCAAGCTCTTGTTCCAGG	CCATCAATGCATCCAAGTCC
CsTEA030725	ATGAGCACCTTGCTGTGTTG	TGGCTGTGTTCGATGTTGCA
TEA016075	GGTGGTCTCTAATGGAGATC	ATGGCGGAAAGGGCAATGAA
EF-1α	GTGTGGAGAAGAAGGACCCA	CGAGGCTAGTGAACAGCAAC

Leaf (TL2)

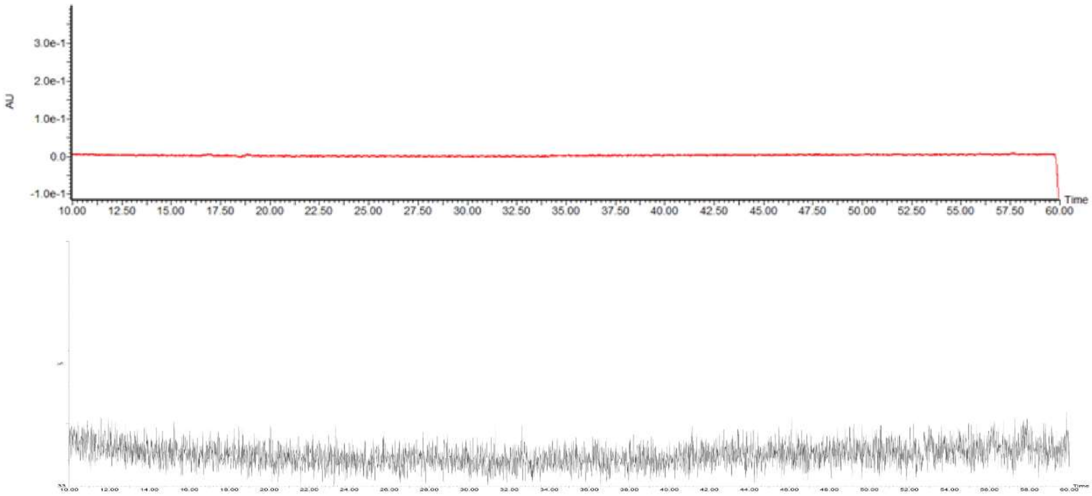

Flower (TF1)

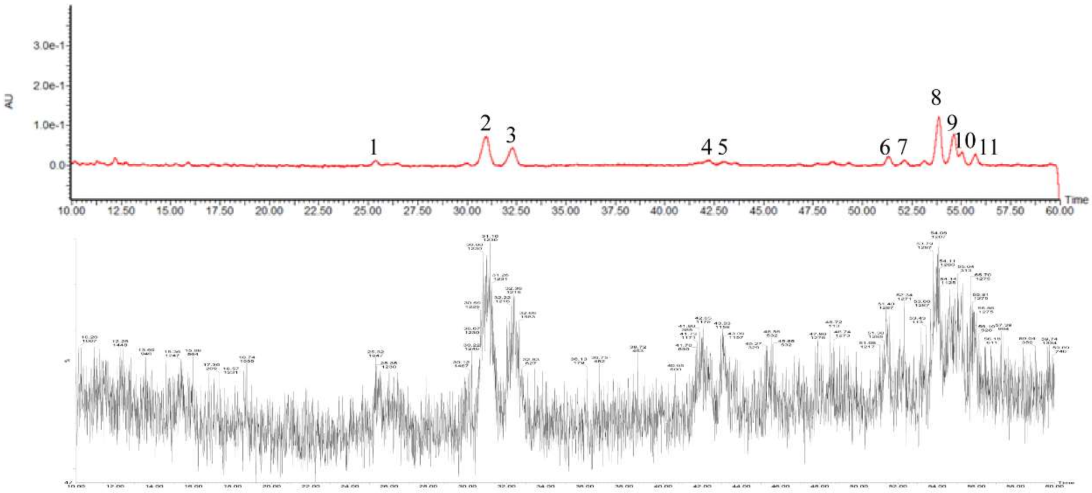

Seed (TS2)

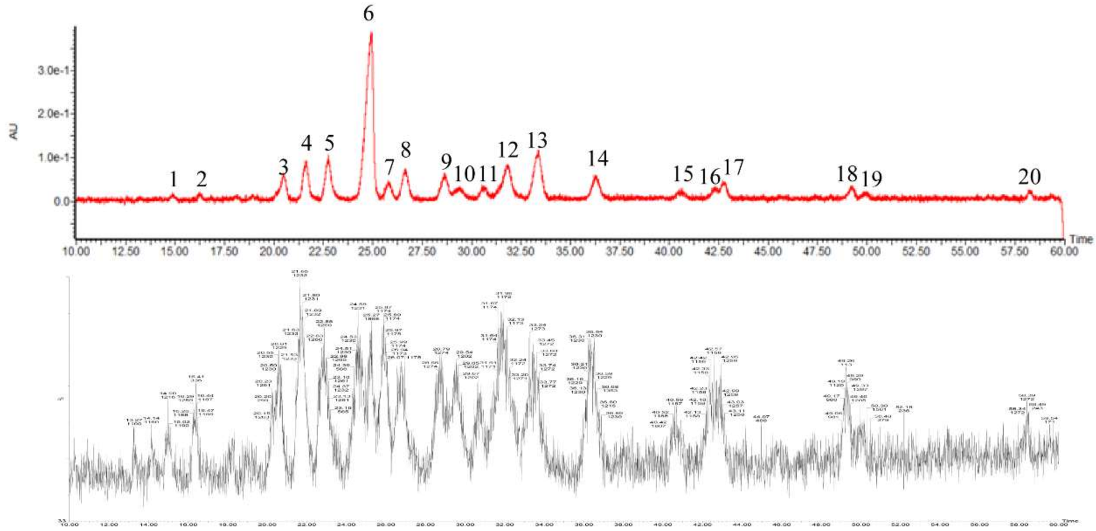

Supplementary Figure S1. Saponins from the leaves, flowers and seeds of *Camellia sinensis* analyzed by UPLC-PDA-QTOF-MS. The red is UPLC-PDA chromatogram at 210 nm, and the black is total ion chromatogram in negative ion mode. Each peak under PDA detector was marked by number, and the individual area of each peak was used to the quantification of saponins in tea flowers and seeds.

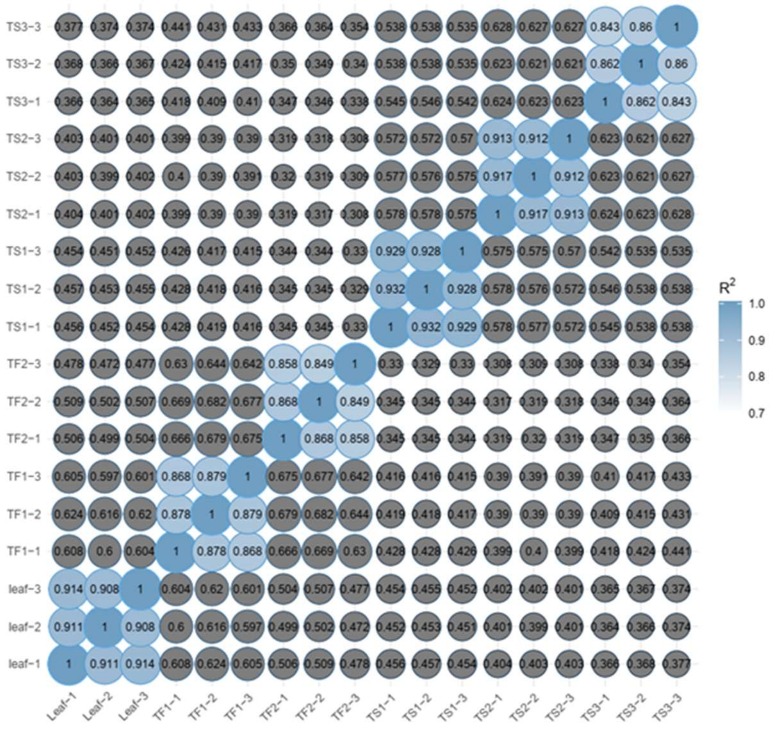

Supplementary Figure S2. Pearson correlation of gene expression among samples.

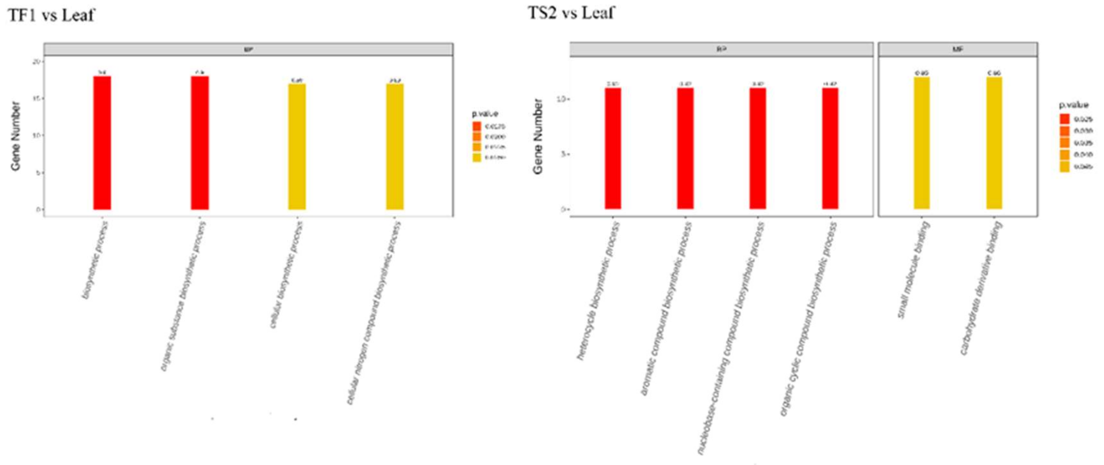

Supplementary Figure S3. Gene Ontology (GO) analysis of differentially expressed genes (DEGs) in two comparison groups including TF1 vs Leaf and TS2 vs Leaf.
